# Elevated Plasma DISC1 Levels Are Associated With Motivational Deficits in Medication‐Free Patients With Major Depressive Disorder: An Exploratory Study

**DOI:** 10.1002/pchj.70123

**Published:** 2026-09-13

**Authors:** Zhenhua Zhu, Xuyuan Yin, Jia Huang, Yi Wang, Qi Qi, Peijie Wang, Simon S. Y. Lui, Li Hui, Raymond C. K. Chan

**Affiliations:** ^1^ Neuropsychology and Applied Cognitive Neuroscience Laboratory, State Key Laboratory of Cognitive Science and Mental Health, Institute of Psychology Chinese Academy of Sciences Beijing China; ^2^ Department of Psychology University of Chinese Academy of Sciences Beijing China; ^3^ Research Center of Biological Psychiatry, Suzhou Guangji Hospital Suzhou Medical College of Soochow University Suzhou China; ^4^ Department of Psychiatry, School of Clinical Medicine The University of Hong Kong Hong Kong China

**Keywords:** CAINS, DISC1, major depressive disorder, motivation and pleasure, plasma biomarker

## Abstract

Disrupted‐in‐Schizophrenia 1 (*DISC1*) has been implicated in major psychiatric disorders, but the relationship between circulating DISC1 protein and dimensional motivation and pleasure‐related features in major depressive disorder (MDD) is unknown. This exploratory cross‐sectional study compared plasma DISC1 levels between 55 medication‐free patients with MDD and 55 healthy controls (HCs) and examined associations with Clinical Assessment Interview for Negative Symptoms (CAINS) scores. Plasma DISC1 was measured by enzyme‐linked immunosorbent assay, and the CAINS Motivation and Pleasure (MAP) and Expression (EXP) domains were assessed. Patients with MDD had higher CAINS–MAP, CAINS–EXP, and total scores; plasma DISC1 levels were nominally higher after covariate adjustment. In MDD, log_10_‐transformed plasma DISC1 correlated with MAP and total CAINS after FDR correction, but not in HCs; Spearman sensitivity analyses yielded the same domain‐specific pattern. These findings provide preliminary evidence of a modest association between plasma DISC1 and CAINS–MAP scores in medication‐free patients with MDD.

## 1 Introduction

Anhedonia and amotivation are core features of major depressive disorder (MDD), impeding patients' functional recovery (Höflich et al. 2019; Pizzagalli and Roberts 2022; Rizvi et al. 2016). Both features overlap with the motivation‐and‐pleasure (MAP) domain of negative symptoms (Kring et al. 2013; Wehr et al. 2024), which refers to deficits in motivation, pleasure, social engagement, and goal‐directed behavior. The MAP domain and the Expression (EXP) domain (which captures reduced verbal, facial, and gestural expression) together constitute the negative syndrome (Wehr et al. 2024). Although the Clinical Assessment Interview for Negative Symptoms (CAINS) was developed for patients with schizophrenia, it is a reliable measure for the MAP and EXP domains in patients with other psychiatric conditions (Kring et al. 2013; Xie et al. 2018). The two‐factor structure of the CAINS has recently been replicated in patients with MDD and bipolar disorder (Li et al. 2025). Therefore, the CAINS can serve as a useful complementary measure to clinical scales for depressive symptoms, such as the Hamilton Depression Rating Scale (HAMD), by better differentiating the MAP and EXP domains. However, the biological correlates of MAP and EXP domains in patients with MDD remain largely unclear.

The Disrupted‐in‐Schizophrenia 1 (*DISC1*) gene was initially identified in a large Scottish family in which a balanced chromosomal translocation was linked to schizophrenia, bipolar affective disorder, and MDD (Hennah et al. 2006). Genetic research further implicates *DISC1* and *TSNAX‐DISC1* variation in MDD and other affective disorders (Hashimoto et al. 2006; Schosser et al. 2010). *DISC1* encodes a multifunctional scaffold protein involved in neurodevelopmental and synaptic processes relevant to psychiatric disorders (Srikanth et al. 2015; Weng et al. 2018). Evidence from animal studies has shown that disruption of *DISC1* was associated with cellular and behavioral abnormalities related to depressive phenotypes (Kim et al. 2021; Sauer et al. 2015). Recently, plasma DISC1 has been quantified in first‐episode schizophrenia, where lower levels were associated with greater CAINS–EXP ratings (Yang et al. 2026), providing an important precedent for directly examining circulating DISC1 and supporting its association with the CAINS dimensions. However, whether plasma DISC1 reflects central *DISC1* expression or function remains unclear.

Evidence also links *DISC1* dysregulation to depressive‐like behavior and social withdrawal. In schizophrenia, lower peripheral *DISC1* mRNA levels were associated with more severe negative and depressive symptoms (Chen et al. 2022). In preclinical studies, a truncated *DISC1* mouse model showed depressive‐like behavior and reduced prefrontal dopamine D2 receptor expression (Wu et al. 2024), and hispidulin attenuated social withdrawal in isolated mutant *DISC1* mice (Mouri et al. 2020). However, these transcriptomic and animal studies do not directly predict the direction of circulating DISC1 protein changes. Together, they provide a rationale for examining whether plasma DISC1 is related to motivation‐, pleasure‐, and expression‐related dimensions in MDD.

We conducted an exploratory cross‐sectional study in medication‐free patients with MDD and healthy controls (HCs). We compared plasma DISC1 levels between the MDD and control groups and examined their associations with the CAINS ratings. Given the paucity of direct evidence, our analyses were exploratory, and we did not make any directional or mechanistic hypotheses.

## 2 Methods

### 2.1 Participants

This cross‐sectional study included 55 patients with MDD and 55 age‐ and sex‐matched HCs. All participants were recruited from the outpatient psychiatry clinic of Suzhou Guangji Hospital from January 2021 to May 2023. The inclusion criteria for the MDD group were (1) a diagnosis of MDD ascertained using the Structured Clinical Interview for *DSM‐5* by two independent psychiatrists, (2) aged 16–65 years, (3) no current psychiatric disorder other than MDD (e.g., bipolar disorder, schizophrenia, or a substance use disorder), and (4) absence of any antidepressant prescription or psychotherapy in the last 4 weeks. The exclusion criteria included (1) medical diagnosis of autoimmune diseases, cancer, or neurological disorders, (2) pregnancy, (3) lactation, (4) acute infections, (5) history of traumatic brain injury, and (6) intellectual disability. The same eligibility criteria applied to HCs, except that they were required to have no personal or first‐degree family history of psychiatric disorder. This study was approved by the Ethics Committee of Suzhou Guangji Hospital (Approval No. 2020‐006), and all participants provided written informed consent.

### 2.2 Clinical Assessments

Sociodemographic and lifestyle data, such as age, sex, education, body mass index (BMI), marital status, smoking, and drinking, were obtained from medical records. Trained psychiatrists who were blinded to the study hypotheses administered the clinical instruments. The CAINS comprises two domains, namely the MAP (which has nine items assessing anhedonia, asociality, and avolition) and the EXP (which has four items assessing reduced verbal and nonverbal expression). The 24‐item Hamilton Depression Rating Scale (HAMD) and the Hamilton Anxiety Rating Scale (HAMA) were also administered.

### 2.3 Plasma DISC1 Measurement

Whole blood samples were collected after overnight fasting (≥ 8 h) and processed in the laboratory within 2 h after sample collection. In the laboratory, the samples were centrifuged at 3000 rpm for 10 min at 4°C to isolate plasma, and plasma aliquots were stored at −80°C until analysis. Plasma DISC1 levels were measured using a commercially available Human DISC1 enzyme‐linked immunosorbent assay (ELISA) kit (Catalog No. AE47203HU, abebio, China), in accordance with the manufacturer's protocol. The assay sensitivity was 31.25 pg/mL, with a detection range of 62.5–4000 pg/mL. Each participant sample was measured once. The samples from patients with MDD and HCs were analyzed using the same experimental batch on separate assay plates. The same QC sample was included on each plate to monitor potential plate‐to‐plate variability. Samples with concentrations below the lower detection limit or above the assay range were excluded from further analysis. A standard curve was generated in triplicate for each plate. The technician performing the assay was blinded to participant identity and clinical information.

### 2.4 Statistical Analysis

Statistical analyses were performed using R (version 4.4.1), with a two‐tailed significance level set at *p* < 0.05. Continuous variables were analyzed using one‐way ANOVA or Mann–Whitney *U* tests, as appropriate, and categorical variables were compared using Chi‐square (*χ* ^2^) tests. We inspected the distribution of the continuous variables using histograms, *Q*–*Q* plots, and Shapiro–Wilk tests. Plasma DISC1 levels were log_10_‐transformed to achieve normality. The CAINS, HAMA, and HAMD ratings were retained in original units for interpretability. Group differences in plasma DISC1 levels as well as CAINS total and subscale scores (MAP and EXP) between patients with MDD and HCs were assessed using analysis of covariance (ANCOVA), adjusted for age, sex, education, BMI, marital status, smoking, and drinking. No a priori sample size calculation was performed for this exploratory study.

Correlational analyses examined the relationships of log_10_‐transformed plasma DISC1 with the CAINS–MAP, CAINS–EXP, and total CAINS scores separately in the MDD and HC groups. Pearson correlation was used for the primary analyses. Because the CAINS scores were not normally distributed, we used Spearman correlations as a sensitivity analysis. Multiple linear regression models were fitted separately for each group and outcome to examine the adjusted associations with the CAINS scores. The regression models included log_10_‐transformed plasma DISC1, sex, age, education, BMI, smoking, drinking, HAMD, and HAMA scores; illness duration was additionally included in the MDD models. Regression diagnostics included variance inflation factors, residual distributions, homoscedasticity, and influential observations. No problematic multicollinearity was detected (all VIFs < 2.0). Benjamini–Hochberg false discovery rate (FDR) correction was applied within conceptually related analysis families. The group comparisons in the three CAINS scores (MAP, EXP, and total scores) constituted one clinical‐rating family, whereas the single plasma DISC1 group comparison was reported separately as a biological comparison. Within each diagnostic group, the *p* values derived from Pearson and Spearman correlations were each corrected separately across the MAP, EXP, and total CAINS scores. The DISC1 terms in the three regression models for the MAP, EXP, and total CAINS scores constituted a separate family within each diagnostic group.

## 3 Results

### 3.1 Demographic and Clinical Characteristics

Demographic and clinical characteristics of MDD participants and HCs are summarized in Table 1. The two groups were comparable in age (*F* (1,108) = 3.35, *p* = 0.070), sex distribution (*χ* ^2^ = 0.33, *p* = 0.564), and BMI (*F* (1,108) = 0.67, *p* = 0.415). Patients with MDD had fewer years of education than HCs (*F* (1,108) = 21.56, *p* < 0.001, *η* ^2^ = 0.17), and higher HAMD‐24 (*F* (1,105) = 277.44, *p* < 0.001, *η* ^2^ = 0.73) and HAMA scores (*F* (1,105) = 165.38, *p* < 0.001, *η* ^2^ = 0.61).

**TABLE 1:** Demographic and clinical characteristics for HCs and MDD.

Variables	HCs	MDD	*χ* ^2^/*F*	*p*	*η* ^2^
Sex (male/female)	22/33	26/29	0.33	0.564	—
Age (years)	29.75 ± 8.72	26.76 ± 8.35	3.35	0.070	0.03
Education (years)	14.18 ± 1.62	12.40 ± 2.34	21.56	< 0.001	0.17
BMI (kg/m^2^)	22.31 ± 3.19	21.77 ± 3.74	0.67	0.415	0.01
Marital status (unmarried/married/divorced)^a^	28/25/1	33/17/5	4.59	0.101	—
Smoking (nonsmoker/smoker)^b^	50/5	44/10	1.32	0.250	—
Drinking (nondrinker/drinker)^c^	48/6	47/7	0.00	1.000	—
Duration of illness (years)	—	2.86 ± 2.83	—	—	—
Age of onset (years)	—	24.00 ± 8.84	—	—	—
HAMD total score^d^	2.38 ± 3.38	29.09 ± 11.08	277.44	< 0.001	0.73
HAMA total score^e^	1.48 ± 2.02	20.49 ± 10.47	165.38	< 0.001	0.61

*Note:* Data are expressed as mean ± SD for continuous variables. For categorical variables such as sex, data are shown as *n*/*n* (e.g., male/female).

Abbreviations: BMI, body mass index; HAMA, Hamilton Anxiety Rating Scale; HAMD, Hamilton Depression Rating Scale; HCs, healthy controls; MDD, major depressive disorder; SD, standard deviation.

^a^ Marital status data were missing for one participant in the HC group.

^b^ Smoking status data were missing for one participant in the MDD group.

^c^ Drinking status data were missing for one participant in each group.

^d^ HAMD total scores were missing for three participants in the HC group.

^e^ HAMA total scores were missing for three participants in the HC group.

### 3.2 Group Differences in CAINS Ratings

Participants with MDD showed higher CAINS–MAP, CAINS–EXP, and total CAINS scores than HCs (Table 2). Unadjusted analyses revealed pronounced differences in CAINS–MAP (*F* (1,108) = 86.39, *p* < 0.001, *η* ^2^ = 0.44), CAINS–EXP (*F* (1,108) = 83.65, *p* < 0.001, *η* ^2^ = 0.44) and total CAINS score (F (1,108) = 116.29, *p* < 0.001, *η* ^2^ = 0.52). The differences remained statistically significant after adjusting for age, sex, education, BMI, marital status, smoking, and drinking, with large effect sizes for CAINS–MAP (*F* adj (1,98) = 48.29, *p* < 0.001, $\eta_{p}^{2}$ = 0.33), CAINS–EXP (*F* adj (1,98) = 47.97, *p* < 0.001, $\eta_{p}^{2}$ = 0.33), and total CAINS scores (*F* adj (1,98) = 65.50, *p* < 0.001, $\eta_{p}^{2}$ = 0.40). All three comparisons remained significant after FDR correction within the clinical‐rating family (all FDR‐adjusted *p* < 0.001).

**TABLE 2:** Comparisons of the CAINS scores between HCs and MDD.

Variables	HCs	MDD	*F* (1,108)	*p*	*η* ^2^	ANCOVA *F* (1,98)	ANCOVA *p*	$\eta_{p}^{2}$
MAP	7.87 ± 4.85	19.69 ± 8.09	86.39	< 0.001	0.44	48.29	< 0.001	0.33
EXP	0.27 ± 0.80	6.35 ± 4.86	83.65	< 0.001	0.44	47.97	< 0.001	0.33
CAINS total score	8.15 ± 4.89	26.04 ± 11.29	116.29	< 0.001	0.52	65.50	< 0.001	0.40

*Note:* Sex, age, education, BMI, marital status, smoking, drinking. All three group comparisons remained significant after FDR correction (all FDR‐adjusted *p* < 0.001).

Abbreviations: CAINS, Clinical Assessment Interview for Negative Symptoms; EXP, Expression domain of CAINS; MAP, Motivation and Pleasure domain of CAINS.

### 3.3 Plasma DISC1 Protein Levels

**FIGURE 1:**
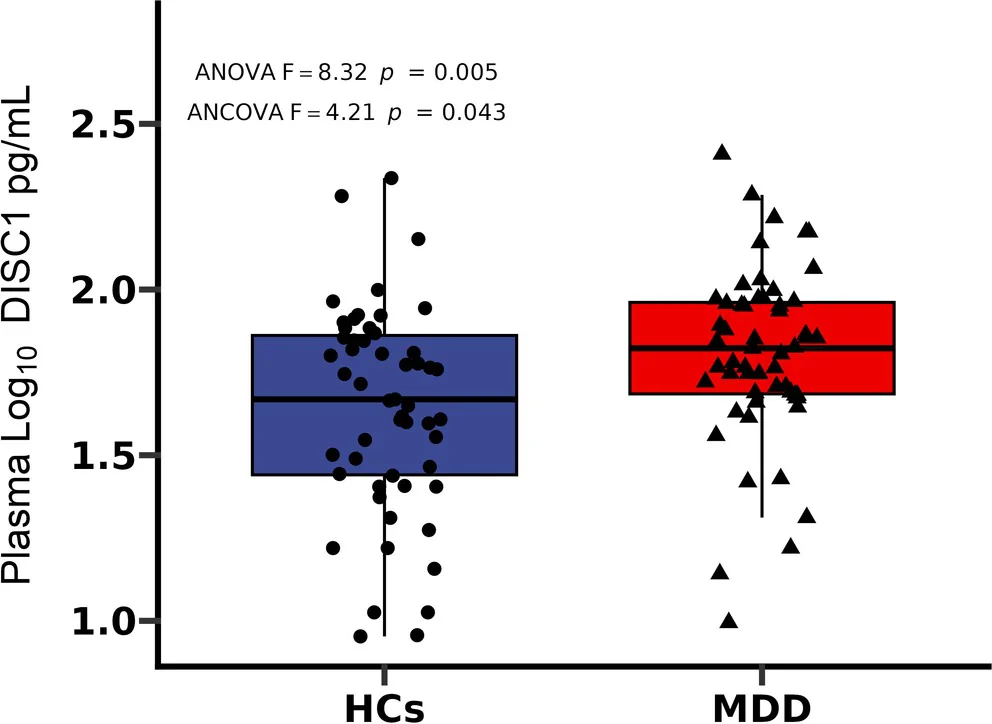
Group comparison of plasma DISC1 levels. Log_10_‐transformed plasma DISC1 levels were higher in patients with MDD (*n* = 55) than in HCs (*n* = 55) in the unadjusted analysis (*F* (1,108) = 8.32, *p* = 0.005) and remained nominally significant after adjustment for age, sex, education, BMI, marital status, smoking, and drinking (*F* (1,98) = 4.21, *p* = 0.043). ANCOVA, analysis of covariance; ANOVA, analysis of variance.

Log_10_‐transformed plasma DISC1 levels were higher in patients with MDD than in HCs in the unadjusted analysis (*F* (1,108) = 8.32, *p* = 0.005) and remained nominally significant after adjustment for age, sex, education, BMI, marital status, smoking, and drinking (*F* (1,98) = 4.21, *p* = 0.043) (Figure 1).

### 3.4 Associations Between Plasma DISC1 and CAINS Scores

**FIGURE 2:**
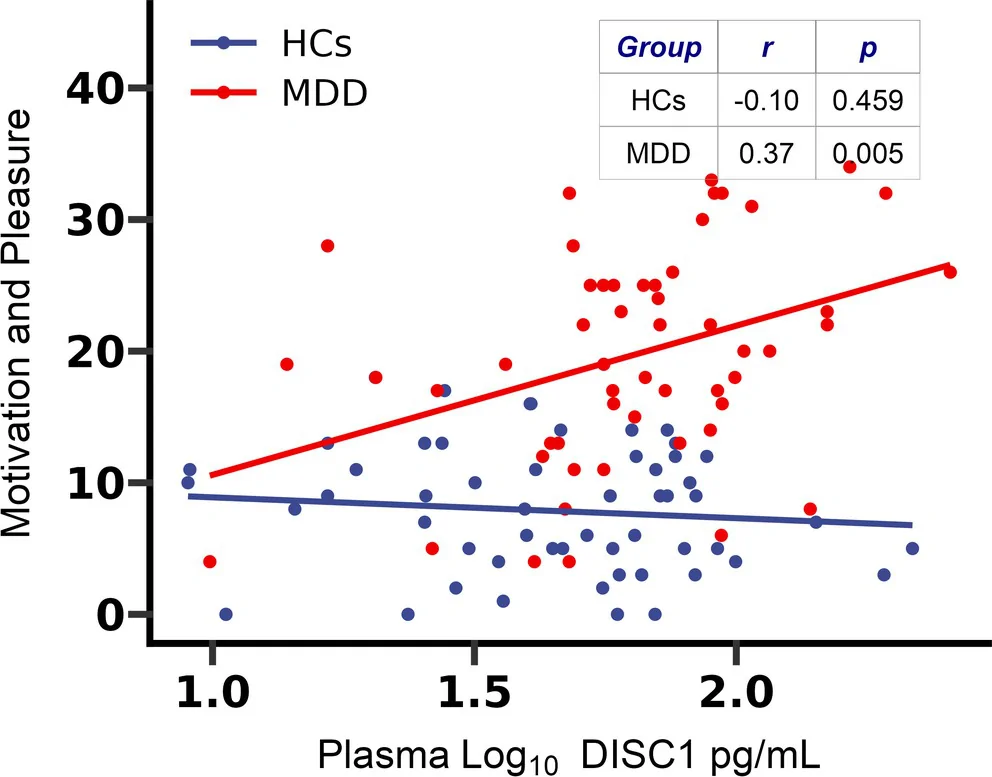
Association between plasma DISC1 and CAINS–MAP scores. In patients with MDD, log_10_‐transformed plasma DISC1 correlated with CAINS–MAP (*r* (53) = 0.37, uncorrected *p* = 0.005, FDR‐adjusted *p* = 0.015), whereas no association was detected in HCs (*r* (53) = −0.10, uncorrected *p* = 0.459, FDR‐adjusted *p* = 0.621).

In patients with MDD, log_10_‐transformed plasma DISC1 levels showed a moderate positive correlation with CAINS–MAP (*r* (53) = 0.37, uncorrected *p* = 0.005, FDR‐adjusted *p* = 0.015) (Figure 2) and total CAINS scores (*r* (53) = 0.30, uncorrected *p* = 0.027, FDR‐adjusted *p* = 0.041) (Figure S1), but not CAINS–EXP (*r* (53) = 0.07, uncorrected *p* = 0.610, FDR‐adjusted *p* = 0.610) (Figure S2). No corresponding correlations were detected in HCs (MAP: *r* (53) = −0.10, uncorrected *p* = 0.459, FDR‐adjusted *p* = 0.621; EXP: *r* (53) = 0.20, uncorrected *p* = 0.141, FDR‐adjusted *p* = 0.423; total CAINS scores: *r* (53) = −0.07, uncorrected *p* = 0.621, FDR‐adjusted *p* = 0.621). Spearman sensitivity analyses yielded the same domain‐specific pattern (Table S3). In patients with MDD, plasma DISC1 correlated with CAINS–MAP (rho = 0.38, uncorrected *p* = 0.004, FDR‐adjusted *p* = 0.012) and total CAINS (rho = 0.35, uncorrected *p* = 0.009, FDR‐adjusted *p* = 0.014), but not CAINS–EXP (rho = 0.11, uncorrected *p* = 0.413, FDR‐adjusted *p* = 0.413). None of the Spearman correlations was significant in HCs (all FDR‐adjusted *p* = 0.530).

In the MDD group, multiple linear regression showed that plasma DISC1 was associated with CAINS–MAP scores before correction (*B* = 8.39, SE = 3.69, *t* = 2.27, *p* = 0.028), controlling for the covariates listed in Table 3. This term did not survive FDR correction across the three regression models (FDR‐adjusted *p* = 0.084). Plasma DISC1 was not associated with total CAINS (*B* = 9.17, *p* = 0.088, FDR‐adjusted *p* = 0.132) or CAINS–EXP (*B* = 0.78, *p* = 0.762, FDR‐adjusted *p* = 0.762) in the adjusted models in the MDD group. The corresponding DISC1 terms were also nonsignificant in HCs (Tables S1 and S2).

**TABLE 3:** Adjusted linear regression models for CAINS–MAP scores in HCs and patients with MDD.

Variable	HCs				MDD			
	*B*	SE	*t*	*p*	*B*	SE	*t*	*p*
Log_10_ plasma DISC1	0.13	2.46	0.05	0.957	8.39	3.69	2.27	0.028
Sex (male/female)	−1.65	1.57	−1.04	0.302	−2.70	1.85	−1.46	0.152
Age (years)	0.06	0.11	0.51	0.612	−0.10	0.11	−0.92	0.365
Education (years)	−0.21	0.56	−0.38	0.708	−0.45	0.39	−1.14	0.259
BMI (kg/m^2^)	0.03	0.24	0.12	0.904	−0.00	0.24	−0.01	0.991
Smoking (nonsmoker/smoker)	−2.06	3.21	−0.64	0.525	−2.98	2.63	−1.14	0.262
Drinking (nondrinker/drinker)	−0.86	2.44	−0.35	0.725	0.66	2.81	0.24	0.814
HAMD total score	0.27	0.40	0.68	0.499	0.59	0.13	4.48	< 0.001
HAMA total score	−0.06	0.67	−0.09	0.928	−0.25	0.14	−1.80	0.078
Duration of illness (years)	—	—	—	—	0.14	0.33	0.43	0.667

*Note:* The *p* values shown are uncorrected. The FDR‐adjusted *p* value for the plasma DISC1 term in the MDD model was 0.084.

## 4 Discussion

To our knowledge, this study is the first to examine plasma DISC1 levels and their associations with CAINS‐defined symptom dimensions in medication‐free patients with MDD. Patients with MDD had higher plasma DISC1 levels in the unadjusted analysis, although the adjusted group difference was only nominally significant. Within the MDD group, higher plasma DISC1 was associated with CAINS–MAP and total scores after FDR correction in both Pearson and Spearman analyses, but not with CAINS–EXP. In the regression models, the DISC1–MAP association was significant before, but not after, correction across the three outcomes. This pattern suggests a modest bivariate association with MAP rather than a robust independent or generalized association across CAINS domains.

*DISC1* is involved in neurodevelopmental and synaptic processes relevant to affective and motivational behavior (Kim et al. 2021; Sauer et al. 2015; Srikanth et al. 2015; Weng et al. 2018). Altered *DISC1* mRNA levels have been associated with negative and depressive symptoms in schizophrenia (Chen et al. 2022). This literature is relevant to the DISC1–MAP association observed here. MAP extends beyond anhedonia to include motivation, social engagement, and goal‐directed behavior. The association may therefore reflect this broader motivation‐ and pleasure‐related dimension. The lack of an association with CAINS–EXP argues against a uniform relationship across CAINS domains. Our prior study in first‐episode schizophrenia found that lower plasma DISC1 levels were associated with greater CAINS–EXP scores (Yang et al. 2026). However, our study differed from prior work in diagnostic groups, symptom distribution, and study design, precluding direct comparison. The relationship between plasma DISC1 and CAINS dimensions may therefore differ across psychiatric populations and symptom domains.

The higher plasma DISC1 levels observed in MDD extend previous evidence linking DISC1 to affective disorders. *DISC1* and *TSNAX‐DISC1* variants have been associated with MDD and other affective disorders (Hashimoto et al. 2006; Schosser et al. 2010), whereas experimental disruption of DISC1 has been linked to neurodevelopmental, synaptic, and depressive‐like phenotypes (Kim et al. 2021; Sauer et al. 2015; Srikanth et al. 2015; Weng et al. 2018). Although the earlier work addressed genetic or experimental alterations rather than circulating proteins, the plasma finding extends this line of evidence to a peripheral measure. All samples in our study were analyzed in the same experimental batch using a standardized protocol, and the same QC sample was included on each plate as a common reference for monitoring plate‐to‐plate assay performance. The group difference supports further study of plasma DISC1 in relation to clinical heterogeneity in MDD.

Patients with MDD also had higher CAINS–MAP, CAINS–EXP, and total CAINS scores than HCs. The higher MAP and total scores are consistent with the established alterations in reward processing, motivation, and pleasure in MDD (Höflich et al. 2019; Pizzagalli and Roberts 2022). The elevated EXP dimension in MDD is more difficult to interpret. Although the MAP/EXP structure has been replicated in MDD (Li et al. 2025), it remains unclear whether elevated EXP scores in patients with MDD have the same clinical and neurobiological meaning as elevated EXP scores in patients with schizophrenia. In MDD, the CAINS may complement the HAMD by better characterizing variation in motivation, pleasure, and expression, rather than only indicating the overall severity of depressive symptoms.

This study has several limitations. First, the sample size was modest for regression models with multiple covariates, and the adjusted DISC1–MAP term did not survive FDR correction. Second, our cross‐sectional design cannot establish temporal direction or causality. Third, participants were not selected using a CAINS threshold, and the education imbalance may have influenced clinical interview performance or symptom reporting, even after covariate adjustment. Fourth, each participant sample was measured once, and the diagnostic groups were assayed on different plates. Finally, the biological origin of plasma DISC1 and its relationship to central DISC1 expression or function remains uncertain.

In this exploratory study, medication‐free patients with MDD had higher plasma DISC1 levels, although the adjusted group difference was nominally significant. Within the MDD group, plasma DISC1 was correlated with CAINS–MAP and total CAINS scores, but not with CAINS–EXP. Larger longitudinal studies are needed to replicate these findings and clarify the biological meaning of circulating DISC1.

## Funding

This work was supported by the National Natural Science Foundation of China (82371508 and 81771439), the Jiangsu Provincial Key Research and Development Program (BE2020661), the Natural Science Foundation of Jiangsu Province (BK20200210), the Jiangsu Provincial Administration of Traditional Chinese Medicine (MS2022083), the Suzhou Municipal Sci‐Tech Bureau Program (SKY2022065), the Suzhou Municipal Health Commission Science Research Program (ZDXM2025019), and the Sample Bank of Suzhou Municipal Psychiatric Disorders with support from the Suzhou Municipal Finance Bureau.

## Conflicts of Interest

The authors declare no conflicts of interest.

## Supporting information


**Table S1:** Adjusted linear regression models for total CAINS scores in HCs and patients with MDD.
**Table S2:** Adjusted linear regression models for CAINS–EXP scores in HCs and patients with MDD.
**Table S3:** Spearman rank‐correlation sensitivity analyses between plasma DISC1 and CAINS scores.
**Figure S1:** Plasma DISC1 levels were moderately positively correlated with total CAINS score in patients with MDD (*r* = 0.30, *p* = 0.027), whereas no significant correlation was observed in healthy controls (*r* = −0.07, *p* = 0.621).
**Figure S2:** No significant association was found between plasma DISC1 levels and expressivity (CAINS–EXP scores) in either patients with MDD (*r* = 0.07, *p* = 0.610) or healthy controls (*r* = 0.20, *p* = 0.141).

## Data Availability

The data that support the findings of this study are available on request from the corresponding author. The data are not publicly available due to privacy or ethical restrictions.
