# Supplementary material for: Elevated Plasma DISC1 Levels Are Associated With Motivational Deficits in Medication‐Free Patients With Major Depressive Disorder: An Exploratory Study
Source: Psych J. 2026 Sep 13;15(5):e70123. doi: 10.1002/pchj.70123 (PMC13571855; doi:10.1002/pchj.70123)
Supplement: Supplementary file 1 [file PCHJ-15-e70123-s001.docx]

**Supplementary Materials**

## S1. Supplementary Tables

Table S1 Adjusted linear regression models for total CAINS scores in HCs and patients with MDD

Table S2 Adjusted linear regression models for CAINS-EXP scores in HCs and patients with MDD

Table S3 Spearman rank-correlation sensitivity analyses between plasma DISC1 and CAINS scores

## S2. Supplementary Figures

Figure S1 Plasma DISC1 levels were moderately positively correlated with total CAINS score in patients with MDD (r = 0.30, *p* = 0.027), whereas no significant correlation was observed in healthy controls (r = –0.07, *p* = 0.621).

Figure S2 No significant association was found between plasma DISC1 levels and expressivity (CAINS-EXP scores) in either patients with MDD (r = 0.07, *p* = 0.610) or healthy controls (r = 0.20, *p* = 0.141).

Table S1 Adjusted linear regression models for total CAINS scores in HCs and patients with MDD

| Variable | HCs | | | |  | MDD | | | |
| --- | --- | --- | --- | --- | --- | --- | --- | --- | --- |
|  | B | SE | t | p |  | B | SE | t | p |
| Log10 plasma DISC1 | 0.28 | 2.51 | 0.11 | 0.913 |  | 9.17 | 5.26 | 1.74 | 0.088 |
| Sex (male/female) | -1.48 | 1.60 | -0.92 | 0.362 |  | -4.41 | 2.64 | -1.67 | 0.102 |
| Age (years) | 0.02 | 0.12 | 0.16 | 0.872 |  | -0.30 | 0.15 | -2.01 | 0.051 |
| Education (years) | -0.31 | 0.57 | -0.55 | 0.588 |  | -0.63 | 0.56 | -1.13 | 0.265 |
| BMI (kg/m^2^) | 0.05 | 0.25 | 0.21 | 0.838 |  | -0.04 | 0.34 | -0.11 | 0.911 |
| Smoking (nonsmoker/smoker) | -1.38 | 3.27 | -0.42 | 0.675 |  | -4.92 | 3.74 | -1.32 | 0.195 |
| Drinking (nondrinker/drinker) | 0.43 | 2.48 | 0.17 | 0.865 |  | -2.78 | 4.00 | -0.70 | 0.491 |
| HAMD Total score | 0.28 | 0.41 | 0.70 | 0.491 |  | 0.74 | 0.19 | 3.97 | <0.001 |
| HAMA Total score | -0.09 | 0.68 | -0.13 | 0.900 |  | -0.33 | 0.20 | -1.68 | 0.101 |
| Duration of illness (years) | - | - | - | - |  | 0.25 | 0.47 | 0.53 | 0.599 |

Table S2 Adjusted linear regression models for CAINS-EXP scores in HCs and patients with MDD

| Variable | HCs | | | |  | MDD | | | |
| --- | --- | --- | --- | --- | --- | --- | --- | --- | --- |
|  | B | SE | t | p |  | B | SE | t | p |
| Log10 plasma DISC1 | 0.14 | 0.35 | 0.41 | 0.688 |  | 0.78 | 2.57 | 0.31 | 0.762 |
| Sex (male/female) | 0.17 | 0.22 | 0.75 | 0.458 |  | -1.70 | 1.29 | -1.32 | 0.193 |
| Age (years) | -0.04 | 0.02 | -2.42 | 0.020 |  | -0.21 | 0.07 | -2.79 | 0.008 |
| Education (years) | -0.10 | 0.08 | -1.26 | 0.215 |  | -0.18 | 0.27 | -0.67 | 0.508 |
| BMI (kg/m^2^) | 0.02 | 0.03 | 0.62 | 0.538 |  | -0.04 | 0.17 | -0.21 | 0.833 |
| Smoking (nonsmoker/smoker) | 0.68 | 0.46 | 1.49 | 0.144 |  | -1.94 | 1.83 | -1.06 | 0.296 |
| Drinking (nondrinker/drinker) | 1.29 | 0.35 | 3.71 | <0.001 |  | -3.44 | 1.95 | -1.76 | 0.085 |
| HAMD Total score | 0.01 | 0.06 | 0.18 | 0.861 |  | 0.15 | 0.09 | 1.69 | 0.099 |
| HAMA Total score | -0.02 | 0.10 | -0.26 | 0.796 |  | -0.08 | 0.10 | -0.84 | 0.405 |
| Duration of illness (years) | - | - | - | - |  | 0.11 | 0.23 | 0.46 | 0.646 |

Table S3 Spearman rank-correlation sensitivity analyses between plasma DISC1 and CAINS scores

| Group | Outcome | Spearman rho | Uncorrected p | FDR-adjusted p |
| --- | --- | --- | --- | --- |
| MDD | CAINS-MAP | 0.38 | 0.004 | 0.012 |
| MDD | CAINS-EXP | 0.11 | 0.413 | 0.413 |
| MDD | Total CAINS | 0.35 | 0.009 | 0.014 |
| HCs | CAINS-MAP | -0.10 | 0.482 | 0.530 |
| HCs | CAINS-EXP | 0.09 | 0.494 | 0.530 |
| HCs | Total CAINS | -0.09 | 0.530 | 0.530 |

Note. Spearman correlations used log10-transformed plasma DISC1. Benjamini-Hochberg FDR adjustment was applied separately within each diagnostic group across CAINS-MAP, CAINS-EXP, and total CAINS. CAINS, Clinical Assessment Interview for Negative Symptoms; MAP, Motivation and Pleasure; EXP, Expression; MDD, major depressive disorder; HCs, healthy controls.

Figure S1 Plasma DISC1 levels were moderately positively correlated with total CAINS score in patients with MDD (r = 0.30, *p* = 0.027), whereas no significant correlation was observed in healthy controls (r = –0.07, *p* = 0.621).

Figure S2 No significant association was found between plasma DISC1 levels and expressivity (CAINS-EXP scores) in either patients with MDD (r = 0.07, *p* = 0.610) or healthy controls (r = 0.20, *p* = 0.141).
